# Mechanical and Microstructural Investigation of Geopolymer Concrete Incorporating Recycled Waste Plastic Aggregate

**DOI:** 10.3390/ma17061340

**Published:** 2024-03-14

**Authors:** Blessing O. Adeleke, John M. Kinuthia, Jonathan Oti, Duncan Pirrie, Matthew Power

**Affiliations:** 1 Faculty of Computing, Engineering and Science, University of South Wales, Pontypridd CF37 1DL, UK; john.kinuthia@southwales.ac.uk (J.M.K.); jonathan.oti@southwales.ac.uk (J.O.); duncan.pirrie@southwales.ac.uk (D.P.); 2 Vidence Inc., 4288 Lozells Avenue, Suite 213L, Burnaby, BC V5A 0C7, Canada; matthew.power@videnceinc.com

**Keywords:** alkali–alkaline activator, GGBS, silica fume, consistency, compressive strength, scanning electron microscopy, waste plastic, aluminosilicate, geopolymer

## Abstract

The effective use of waste materials is one of the key drivers in ensuring sustainability within the construction industry. This paper investigates the viability and efficacy of sustainably incorporating a polylactic acid-type plastic (WP) as a 10 mm natural coarse aggregate (NA) replacement in geopolymer concrete. Two types of concrete (ordinary Portland cement—OPC and geopolymer) were produced for completeness using a concrete formulation ratio of 1:2:3. The ordinary concrete binder control was prepared using 100% OPC at a water/binder ratio of 0.55, while the geopolymer concrete control used an optimum alkaline activator/precursor—A/P ratio (0.5) and sodium silicate to sodium hydroxide—SS/SH volume ratio (1.2/0.8). Using the same binder quantity as the control, four concrete batches were developed by replacing 10 mm NA with WP at 30 and 70 wt% for ordinary and geopolymer concrete. The mechanical performance of the developed concrete was assessed according to their appropriate standards, while a microstructural investigation was employed after 28 days of curing to identify any morphological changes and hydrated phases. The results illustrate the viability of incorporating WP in geopolymer concrete production at up to 70 wt% replacement despite some negative impacts on concrete performance. From a mechanical perspective, geopolymer concrete indicated a 46.7–58.3% strength development superiority over ordinary concrete with or without WP. The sample composition and texture quantified using automated scanning electron microscopy indicated that adding WP reduced the presence of pores within the microstructure of both concrete types. However, this was detrimental to the ordinary concrete due to the low interfacial zone (ITZ) between calcium silicate hydrate (CSH) gel and WP, resulting in the formation of cracks.

## 1. Introduction

Since the 20th century, the global production of plastics has significantly increased from approximately 240 million tonnes in 2002 to 299 million tonnes in 2013 and is continuously rising [1]. For instance, 359 million tons of plastic were produced in 2018, with 51% coming from Asia and 17% from Europe [2]. According to the Association of Plastic Manufacturers—PlasticsEurope [3] and Brahney et al. [4], the current rate of plastic production was estimated at 370 million metric tons per year; at that rate, about 11 billion metric tons of plastic will have accumulated in the environment by 2025. In India alone, the annual waste plastic production is currently at 40 million tonnes, increasing at a rate of 1.5% to 2% yearly [1]. 

The construction industry has also resulted in waste plastic (WP) generation with the constant need for new infrastructure worldwide [5]. Unfortunately, this has resulted in a high demand for construction materials which have contributed significantly to waste production, and this was identified as one key factor in the construction industry contributing to pollution worldwide [6]. The negative environmental implications of WP, as corroborated by Zhang et al. [7] and Xiang et al. [8], cannot be overemphasised as it disintegrates into micro-plastics (MPs, 1 μm–5 mm) and nano-plastics (NPs, 1 nm–1000 nm) in nature due to ultraviolet (UV) radiation, physical abrasion, biological metabolism, mechanical, and temperature changes, and the resultant micro-plastics impact on water and soil quality. Furthermore, micro- and nano-plastics can be transported through aeolian and aqueous processes. They possess an enhanced capability to adsorb and transfer other environmental pollutants, such as heavy metals and organic contaminants [9]. Despite the high contamination potential of WP at the finest particle level, Oliveira et al. [10] and Bridson et al. [11] further described WP as a worrying environmental problem resulting from the leaching of hazardous substances used in producing polymer products, such as lubricants, surfactants, solvents, flame-retardant catalysts, antioxidants, plasticizers, and colourants. Hence, all these industrial products potentially pose significant risks to animal and human health in the form of undesirable immune responses, acute reactions, cytotoxicity, and hypersensitivity [12,13,14].

The increased production of WP does, therefore, require effective strategies to be adopted and implemented to reduce the potential negative health and environmental impacts. These strategies include recycling/reusing using mechanical, biological, or thermochemical techniques, incineration for energy recovery, and landfilling [1,6]. However, only 9% of waste plastic is recycled worldwide, while 80% is disposed in landfill or dispersed in both terrestrial and aquatic ecosystems [6,7,15]. Additionally, the incineration approach is not sustainable as it involves a combustion process that emits carbon dioxide (CO_2_) gas along with potentially toxic elements into the atmosphere. Geyer et al. [16] and Yang et al. [17] also agreed that incineration does not entirely destroy WP since the bottom ash can contain remnants of partly burnt waste (micro-plastics), which is known to be a potential pollution source.

The application of waste in the manufacturing of construction materials is broadly acknowledged as an effective approach to limit the harmful consequences of WP and potential arsenic toxicity [18]. Past studies have shown that reusing waste materials has become a widespread strategy in developing and developed countries to reduce the environmental impacts of waste materials [19,20]. As such, applying WP as an aggregate in construction materials is a reasonable reuse/recycling strategy that promotes environmentally friendly and green construction by substituting these wastes, as aggregates and fibres, for natural raw materials [1,2]. This approach involves incorporating waste materials (including arsenic-containing wastes) into the construction process, thus preventing their dispersal and to mobilise potential risks to health and the environment. This effectively mobilises the toxic substances within a solid matrix and reduces the chances of releasing them into the environment [18,21]. Previous studies investigated the use of WP in ordinary concrete that used plastic in two forms: plastic fibres or aggregates [1,2,22,23]. In those instances, a complete or partial natural aggregate’s replacement with WP resulted in natural resource savings. After the initial grinding and sizing of WP, granules below the size of 4 mm are employed as fine aggregates, while the fibres are used as additives. Also, roughly crushed WP above 5 mm was used to replace coarse aggregates [6,24]. In terms of lower density, unit weight and thermal conductivity, building materials incorporating WP have demonstrated suitable material properties compared to building materials without WP replacement. However, there is deterioration of the mechanical properties of building materials as the WP dosage increases [24]. Examples of such applications include their incorporation in building brick, paver block, concrete, bitumen and asphalt mixtures, etc. [6].

Compared with traditional ordinary Portland cement (OPC) concrete, the high-strength geopolymer concrete relies on the use of sodium silicate and possesses the additional benefits of reduced CO_2_ emissions and energy consumption [25]. Geopolymers are inorganic alumino-silicate polymers with a three-dimensional network, which are obtained using the reaction of aluminosilicate-rich materials obtained from industrial, municipal, and agricultural wastes and by-products such as fly ash, ground granulated blast-furnace slag (GGBS), rice husk ash, etc., with a highly alkaline aqueous solution [26,27]. This reaction is an alkali activation process where the final products of geopolymerisation are usually manipulated by the chemical composition of the alkaline activators and source aluminosilicate materials that acts as a precursor [28]. Provis [29] also described *“Geopolymers as a form of a subset within the broader alkali-activated binder family. These low-calcium binding materials result from the reaction of an aluminosilicate powder (precursor) with an alkaline activator, typically composed of hydroxide, silicate, carbonate, or sulfate. This reaction leads to the formation of an amorphous aluminosilicate gel and secondary nanocrystalline zeolite-like structures.*” Hama and Hilal [30] investigated the effect of partially replacing sand with plastic waste in self-compacting OPC concrete. The range of compressive strength values was about 65 MPa to 37 MPa and that a decrease in compressive strength was observed as the plastic content increased in comparison with the control mix without WP. The reason for this observation was that the plastic waste was a soft material compared with natural aggregate. Also, Pacheco-Torgal [31] investigated the impact of using polyethylene terephthalate (PET) from plastic bottles as a partial substitute for sand within the OPC concrete with up to 50% plastic replacement. The research concluded that the recycled PET could be substituted into eco-friendly concrete at specific replacement rates. A reason for this conclusion was that by replacing sand with PET, the self-weight of concrete is decreased, making the material more suitable for non-structural elements that do not require high compressive strength. Shaikh [32] reinforced OPC, OPC-fly ash-based composites, and an ambient-cured geopolymer using recycled PET (e.g., polypropylene fibre of length 12–19 mm). This improved the compressive strength of geopolymer composites better than other cementitious composites to ~57 MPa and ~47 MPa when 1.0 vol% and 1.5 vol% PET fibres were added, respectively.

Geopolymer concretes composed of fly ash, blast-furnace slag, and PET waste (bottles) decreased the compressive and tensile strength compared with the control mix without plastics from 20.5 MPa to 13.8 MPa and from 2.7 MPa to 1.2 MPa, respectively. In turn, the 10% replacement of sand with plastic granules as fine aggregate contributed to a slight improvement in the mechanical properties [33]. Lazorenko et al. [6] employed the use of WP as fillers by assessing sustainable construction using novel geopolymer composites by integrating waste plastic of different sizes and shapes. The results indicated that geopolymer composites with fine PET particles showed high strength, while increasing the size of PET particles decreased the workability of the geopolymer. Another example of an existing outcome obtained from replacing certain components in concrete composition with plastic is from Ahmed et al. [2], who investigated geopolymer concrete composites incorporated with recycled plastic aggregates and modified with nano-silica. The authors suggested an optimal use of 50% replacement levels of recycled plastics as a replacement for fine aggregates in geopolymer concrete. 

Notably, little or no research has been conducted to explore the application of WP as a coarse aggregate in geopolymer concrete using either a commercial or laboratory-synthesised silica fume (SF)-derived sodium silicate (SS) solution. Thus, this paper aims to evaluate the viability and efficacy of sustainably incorporating WP as a 10 mm NA replacement in geopolymer concrete production while assessing the mechanical and microstructural performance of the produced geopolymer. The current research findings aim to add to the existing knowledge on geopolymer concrete research, encourage more eco-conscious practices in geopolymer concrete production, whilst promoting a greener and more sustainable future in the construction sector.

## 2. Materials

This study utilised ordinary Portland cement (CEM-II/B-V 32.5R), silica fumes (SFs), sodium hydroxide (NaOH), ground granulated blast-furnace slag (GGBS), aggregates, and waste plastics (WP). OPC was manufactured and supplied by Lafarge Cement UK in accordance with BS EN 197—1:2011 [34]. GGBS was exploited as an aluminosilicate precursor material in this study and was manufactured in accordance with BS EN 15167-1:2006 [35] by Civil and Marine Slag Cement Ltd., Llanwern, Newport, UK.

**Figure 1:**
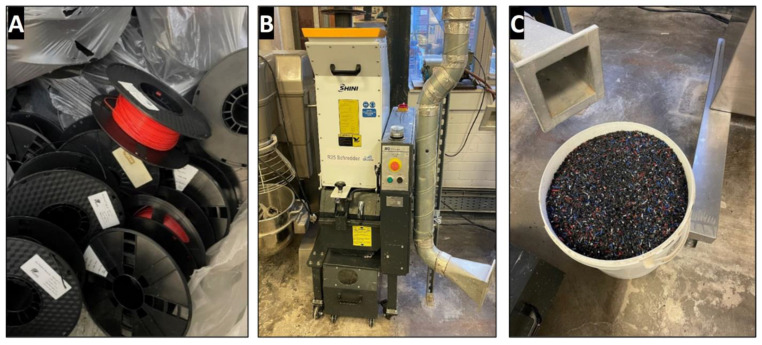
Production of the plastic waste material: (**A**) plastic waste; (**B**) plastic shredder; and (**C**) shredded plastic granules.

**Figure 2:**
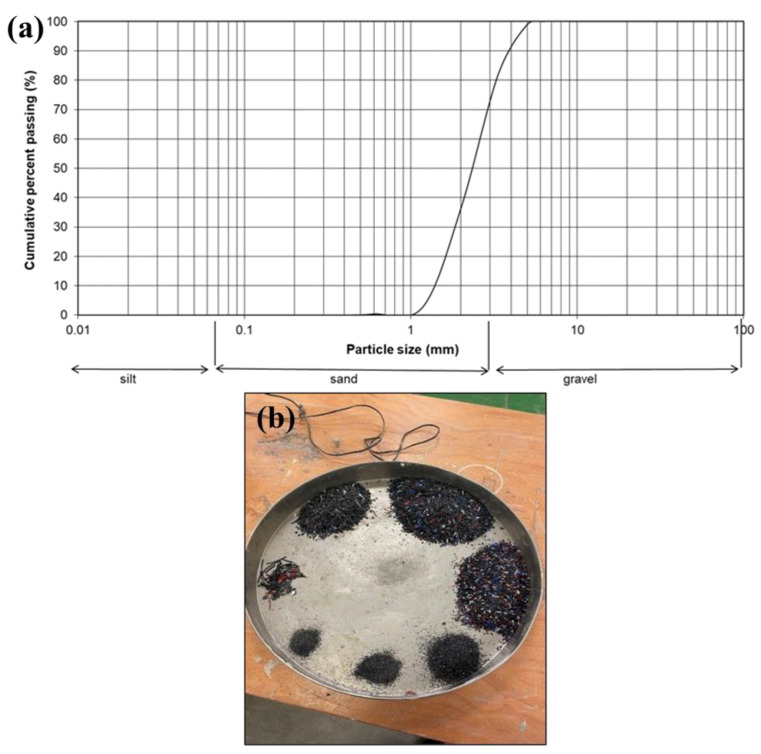
(**a**) Particle size distribution, and (**b**) sieve analysis samples.

The SF was a light-grey amorphous reactive micro-silica powder that was manufactured in Norway by Elkem Silicon Materials and supplied under the trading name of Elkem Un-densified Micro-silica 971 by Tarmac Cement and Lime Company, Buxton Lime and Powders, Derbyshire, Derby, UK. NaOH was used in the form of white laboratory-grade pellets, with a molecular weight of 40 g/mol, pH value of 14, and was commercially sourced from Fisher Scientific Ltd., Loughborough, Leicestershire, UK. The pellets were to produce a sodium hydroxide solution of 10 M [5]. The solution was then used as an activator to dissolve SF to create a sodium silicate solution with SiO_2_/Na_2_O molar ratio of 2. The coarse aggregates are limestone aggregates (20 mm and 10 mm), while the fine aggregate was a natural river sand dredged from the Bristol Channel, UK. A local supplier supplied both coarse and fine aggregates according to the requirements of BS EN 12620:2002+A1:2008 [36]. The WP is a polylactic acid-type plastic used in construction as insulation, furnishing and fibre within carpets, food packaging, and 3D printing [37]. The plastic material used for this research was waste 3D printer filaments broken down using a plastic shredder with the capacity to produce plastic granules between 0–10 mm (see Figure 1). In addition, a particle size distribution curve for the shredded WP between a range of 1–4 mm and sieved samples are shown in Figure 2 according to BS EN ISO 17892-4 [38]. Table 1 shows the chemical oxide compositions for OPC, GGBS, and SF, while Table 2 and Table 3 present their physical properties. Also, Table 3 presents some physical properties of the aggregates (coarse and fine) and WP according to the relevant standards.

## 3. Methodology

### 3.1. Mix Design

The viability and efficacy of sustainably incorporating WP in geopolymer concrete production were assessed in this study. Two controls (C0 and GC0) for ordinary and geopolymer concrete mixes were adopted for completeness as described in the mix design (see Table 4). Mix C0 was prepared with 100% OPC using a water/binder ratio of 0.55, while GC0 was developed in accordance with Adeleke et al. [5] to produce an optimum geopolymer mix by activating GGBS with a binary activator blend of sodium silicate (SS) and sodium hydroxide (SH) solution (1.2/0.8) at an activator/precursor (A/P) mass ratio of 0.5. Using the same binder quantity as the control, other concrete batches were developed by replacing 10 mm NA with WP at 30 and 70 wt% for both ordinary (CWP1 and CWP2) and geopolymer (GCWP1 and GWPC2) concrete using a correction factor of 0.38. The correction factor is the ratio of plastic to aggregate used in the concrete mix according to each percentage replacement level (30 and 70 wt%), allowing the aggregate volume to be replaced instead of its mass. It is worth noting that the concrete mix ratio for all the mix codes were fixed as a binder–sand–aggregates ratio of 1:2:3, and they only vary in terms of the water content, type of binder, and WP replacement levels.

### 3.2. Concrete Specimen Preparation and Testing Methods

The same alkali–alkaline activator (10 M), test sample preparation (geopolymer concrete), and testing methods as in Adeleke et al. [5] were used in this study to produce fresh concrete. A range of tests was conducted, including a slump test (S), unconfined compressive strength (UCS), and automated scanning electron microscopy with linked energy dispersive spectrometers (SEM-EDS). The consistency of the fresh concrete was measured using the slump test (S) in accordance with BS EN 12350-2:2019 [39]. For each batch mix, nine cube (100 mm × 100 mm × 100 mm) test specimens were developed according to BS EN 206:2013+A2:2021 [40]. The geopolymer concrete specimens were placed in a sealed environment (plastic film) to prevent evaporation and kept for 24 h at 20 ± 2 °C. After that, all hardened test specimens were de-moulded after 24 h, while the geopolymer specimens were cured in a sealed container and the ordinary concrete specimens were soaked in water at 20 ± 2 °C and a humidity of 90% [41].

The mechanical performance of the hardened concrete was evaluated in terms of UCS according to BS EN 12390-3:2019 [42] at 7, 28, and 56 days of curing. Lastly, a compositional and microstructural investigation was employed on some of the prepared test specimens after 28 days of curing using the SEM-EDS [43,44]. Representative subsamples were prepared as polished 30 mm diameter blocks which were carbon coated prior to the analysis. A Hitachi SU3900 scanning electron microscope manufactured by Hitachi High-Tech Corporation, Maidenhead, UK, fitted with two large-area (60 mm^2^) Bruker SDD energy dispersive spectrometers and running the Bruker AMICS automated mineralogy package was used to undertake the analysis at an accelerating voltage of 20 kV coupled with a beam current of approximately 10 nA which was used. ED spectra were acquired at a 10 µm interval across the area analysed, and each EDS spectra was compared with a spectral library of known minerals and compositions allowing each analysis spot to be assigned to a compositional grouping. Data outputs from automated SEM-EDS mineral analysis include quantitative modal mineralogy along with false-colour compositional maps and full-area SEM-BSE images. Although plastics are not routinely measured during automated mineralogy analysis, post-processing of the images allowed the plastics to be delineated and quantified. The morphology of representative samples was also interactively examined using an MIRA 3 TESCAN scanning electron microscope.

## 4. Results and Discussions

### 4.1. Consistency of Fresh Concrete

**Figure 3:**
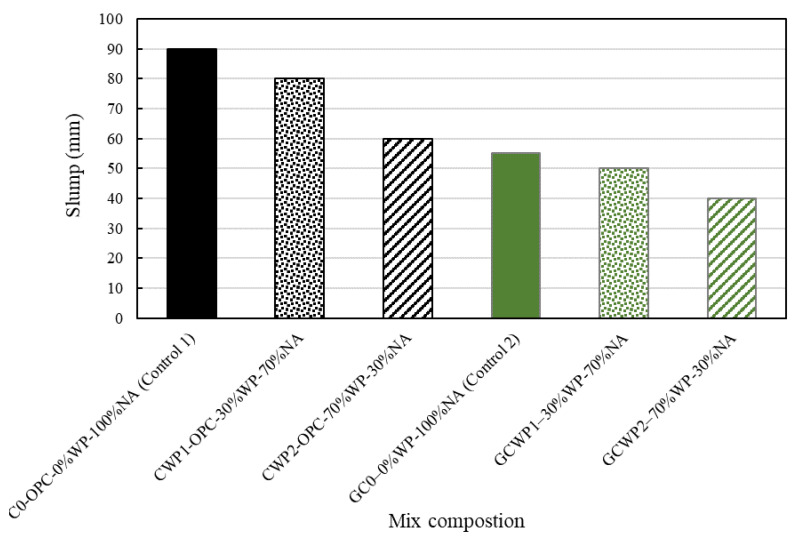
Consistency of concrete mixes measured—slump test.

Figure 3 illustrates the consistency results obtained for the slump. The general trend indicates a lower slump for the geopolymer concrete (GC0, GCWP1, and GCWP2) compared to the ordinary concrete (C0, CWP1, and CWP2). Furthermore, the ordinary concrete has the highest slump values within a 60–90 mm range, while the geopolymer mixes experienced a slump within the 40–55 mm range. The disparity in slump (geopolymer and traditional concrete) could be attributed to the viscosity/quantity of the liquid employed in the concrete mixes and the varying kinetic reactions in each system [5,22]. In addition, GGBS decreases water demand in ordinary concrete mixes as a result of its smooth round particles and lower water absorption; therefore, its use in large quantities within the geopolymer mixes could have negatively impacted the slump values [45].

Furthermore, observation showed that for both the geopolymer and ordinary concrete mixes, there is a decline in slump values for every increase in the quantity of WP. For instance, there was an 11% and 25% decline in slump with a WP quantity of 30 and 70% for the ordinary concrete, while a 9% and 20% decline was observed in the geopolymer concrete at 30 and 70% WP dosage. This shows that any WP replacement of more than 70% could impact the overall workability of the concrete produced. The justification for this slump reduction could be attributed to the non-uniformity in the shape of WP, which could have improved the cohesiveness within each concrete batch mix [23]. In contrast, the 10 mm NA with a more spherical/regular shape enhances the homogeneity and fluidity of the freshly produced concrete mixes. 

A similar finding of decreasing workability of geopolymer mixtures with the inclusion of waste plastic aggregate as a replacement for fine sand was reported by Lazorenko et al. [6], where the authors demonstrated that the inclusion of waste plastic aggregates reduced the workability of the fly ash-based geopolymer mortars when they introduced shredded flakes and strips and ground particles of different sizes to the geopolymer system. Ahmed et al. [2] also indicated that due to the lower specific gravity of recycled plastics than the other elements within a geopolymer mix, they form a structure that resembles a mesh, keeping them together (coagulation) and preventing the concrete mixture from flowing, thus impacting on the overall consistency. Overall, it can be suggested from the consistency results and BS EN 206:2013+A2:2021 [40] that all the concrete formulations can be classified as S2 standard mix, except GCWP2, which is classified as an S1 dry mix.

### 4.2. Concrete Strength Development

**Figure 4:**
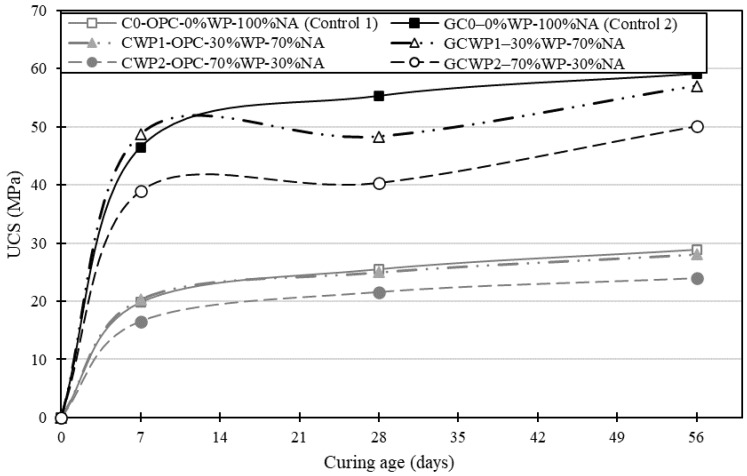
UCS development of the ordinary and geopolymer concrete mixes made with different AP and SS/SH ratios.

Figure 4 illustrates the unconfined compressive strength (UCS) development for all the concrete mixes (ordinary—C0, CWP1, CWP2, and geopolymer—GC0, GCWP1, GCWP2) at 7, 28, and 56 days of the ambient curing period. Observation showed a steady increase in strength development for all of the formulated geopolymer and ordinary concrete mixes over the moist curing periods. In addition, mix C0 and GC0 achieved the highest UCS of 25.5 MPa and 55.4 MPa, respectively, while mix CWP2 and GWP2 with 70% WP replacement levels, performed the lowest with a UCS of 21.5 and 40.4 MPa at 28 days. The strength gain by the ordinary concrete mix can be attributed to the initial hydration of the cement components with water to produce cementing gels (C-S-H), which are required for strength development. In contrast, the trend of strength development for the geopolymer concrete can be attributed to a polymerisation process that involves the chemical reaction of aluminosilicate minerals (GGBS) under alkaline conditions (SS/SH solution), creating a three-dimensional amorphous aluminosilicate matrix that displays strength comparable or superior to ordinary concrete [27,28,46].

**Figure 5:**
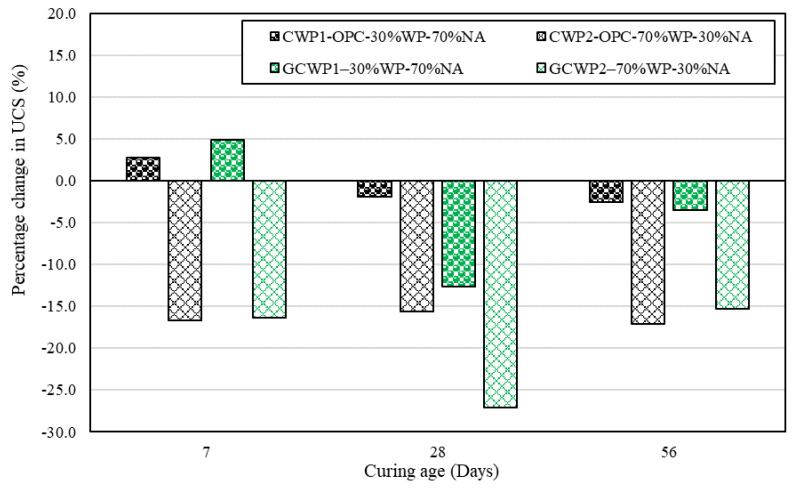
Percentage change in the UCS of each type of concrete formulation (ordinary and geopolymer) compared with their respective controls.

Figure 5 shows the percentage change in the UCS of each type of concrete formulation (ordinary and geopolymer) compared with their respective controls. Observations indicated that replacing 10 mm coarse aggregates with WP at a 30% dosage improved the UCS for both ordinary (2.8%) and geopolymer concrete (4.8%). In contrast, a strength reduction of 16.7% and 16.3% for both ordinary and geopolymer concrete was identified at 7 days of moist curing. A similar trend of declining UCS was observed for all the concrete formulations after 28 and 56 days of moist curing. For instance, the mix of GCWP2 with 70% WP experienced the largest reduction in UCS, while the mix of CWP1 with 30% WP displayed the lowest UCS reduction compared with the control at both 28 and 56 days.

Overall, the UCS reduction for both concrete formulations with the addition of different dosages of WP could be attributed to the lower elastic modulus of WP compared with natural 10 mm coarse aggregate. In addition, it is worth remembering that plastic is considered a hydrophobic material, meaning less water is required for hydration during curing operations [47]. Therefore, the observed decrease in UCS can be linked with the hydrophobic nature and surface smoothness of plastic aggregates, which adds to weak interfacial bonding between the plastic filler (relatively less-rough surfaces of the WPs) and the geopolymer binder matrix. This behaviour is also typical for OPC-based composites incorporated with plastic aggregates [6,24].

Despite some reduction in UCS for the geopolymer concrete when compared with the control, a continuous trend of UCS strength increase can be seen in Figure 4 over the curing age of 7, 28, and 56 days of moist curing. Ahmed et al. [2] attributed this phenomenon to the continuation of the polymerisation process, which enhances the entire geopolymer structures, and, therefore, zones of interfacial transition between WP surfaces and the geopolymer matrix system improve. This behaviour is also typical for ordinary concrete with continuous cement hydration over the curing period.

**Figure 6:**
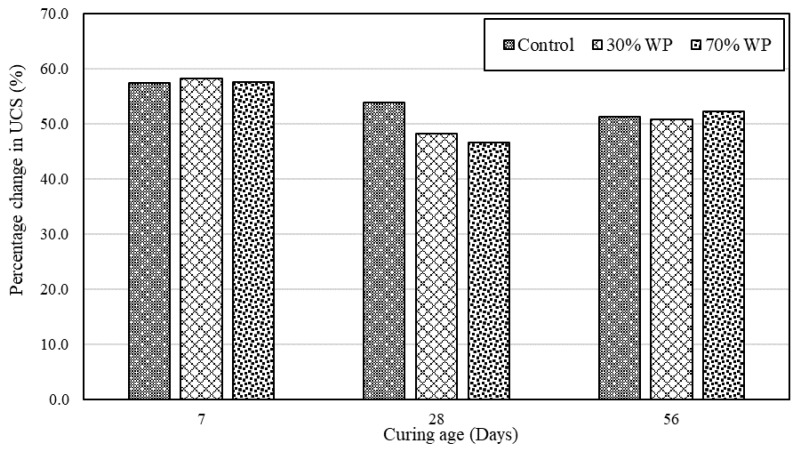
UCS development comparison between geopolymer and ordinary concrete.

Furthermore, a UCS comparison between geopolymer and ordinary concrete as illustrated in Figure 6 showed that despite the inclusion of WP, the geopolymer concrete indicated significant strength development at each curing age duration, indicating the robustness of geopolymer concrete. Moreover, the variation in UCS for the observed concrete mix formulation at each curing age could be due to the varied mix composition incorporating WP at 30 and 70% WP, which impacts the alkali activation/polymerisation process and cement hydration of the developed concrete systems.

### 4.3. Compositional and Microstructural Analysis

The area % quantitative composition of the ordinary and geopolymer concretes with 0, 30, and 70% waste plastics are shown in Table 5, Table 6 and Table 7 for 7-, 28-, and 56-day curing periods, respectively. The quantitative SEM-EDS analysis reports the relative abundance of both the natural aggregate phases (as their constituent minerals), plastics, and the cementitious phases reported as chemical compositional groups. It should be noted that these modal abundance data relate to the 30 mm diameter areas analysed and as such may not be representative of the modal abundance by volume especially as there are coarse aggregate grains present. The OPC reports to the Ca silicate categories, whilst the geopolymer binding reports to the Ca silicate Na, Mg, Al compositional group.

**Figure 7:**
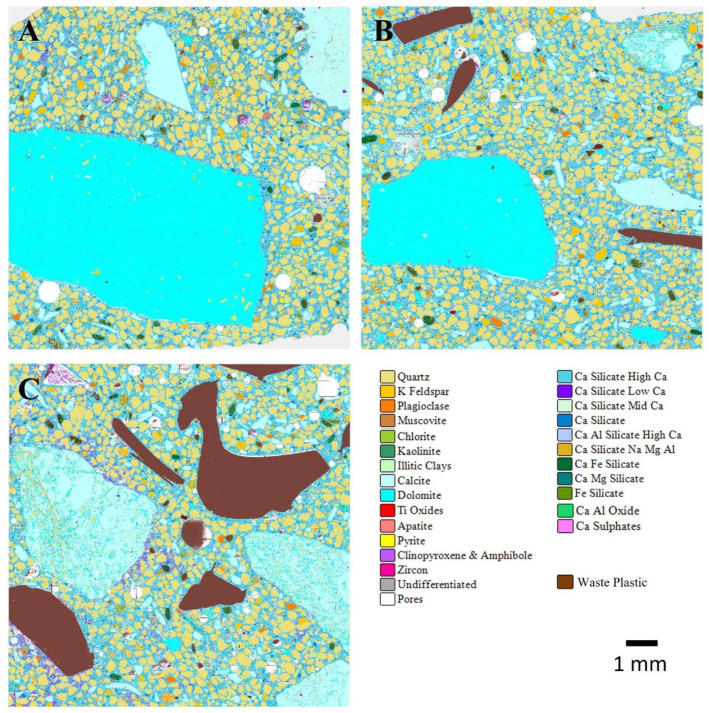
AMICS automated mineralogy false-colour images of 7-day-cured ordinary concrete mixes for (**A**) C0—control, (**B**) CWP1 with 30% WP, and (**C**) CWP2 with 70% WP.

**Figure 8:**
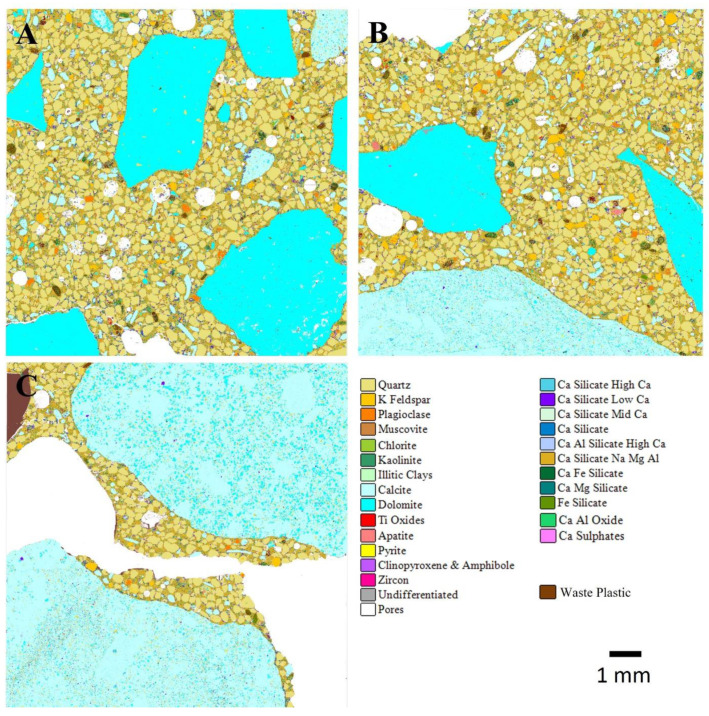
AMICS automated mineralogy false-colour images of 7-day-cured geopolymer concrete mixes for (**A**) GC0—control, (**B**) GCWP1 with 30% WP, and (**C**) GCWP2 with 70% WP.

**Figure 9:**
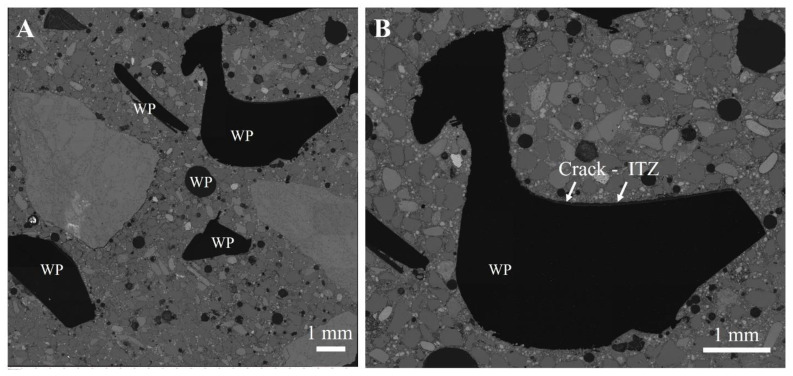
SEM-BSE images of 7-day-cured ordinary concrete mixes for CWP2 with 70% WP. (**A**) Whole area measured; (**B**) enlarged area showing a crack at the margin of the waste plastic (WP).

Figure 7 and Figure 8 show the AMICS automated SEM-EDS images from sections of the ordinary and geopolymer concrete cube specimen at 0%, 30%, and 70%WP at age 7 days of ambient curing. The images show that the ordinary and geopolymer concrete morphology is visibly different, confirming a different type of microstructure after the initial 7-day curing period. Furthermore, visual inspection indicates the presence of pores (see Figure 7 and Figure 8) which are more evident in the control mixes (C0 and GC0) but are gradually reduced with each replacement level of WP in both the ordinary and geopolymer concrete. It is possible that the added WP acted as fillers due to their non-uniformity in shape and size (5–10 mm). However, the ordinary concrete mix of CWP1 and CWP2 matrixes were loose due to cracks between the boundaries of the added WP and the CSH gel formation (Figure 9). Shilar et al. [48] described these boundaries as the interfacial zone (ITZ) between the cementitious hydrate paste and aggregate and are widely known as the weakest link in ordinary concrete, where microcracks typically appear first under stress. The presence of the cracks could be due to the low bonding of the cementitious hydrate gel (CSH) with the WP, which explains the low compressive strength depicted in Figure 4 and Figure 5.

In contrast to the ordinary concrete mix, the presence of pores in the geopolymer mix of GC0, GCWP2, and GCWP2 did not significantly impact the UCS due to the robustness of the geopolymer gel (Na-bearing aluminosilicates) bonding with both the WP and the natural aggregates. Ahmed et al. [2] supported this claim by stating that the use of NaOH-activated sodium silicate enhances the creation of an aluminium-enriched aluminosilicate surface on the aggregates (WP and natural) by speeding up the Si-preferential dissolution of kaolin and albite, thus, increasing the ITZ bonding strength between the aggregates and geopolymer gel.

**Figure 10:**
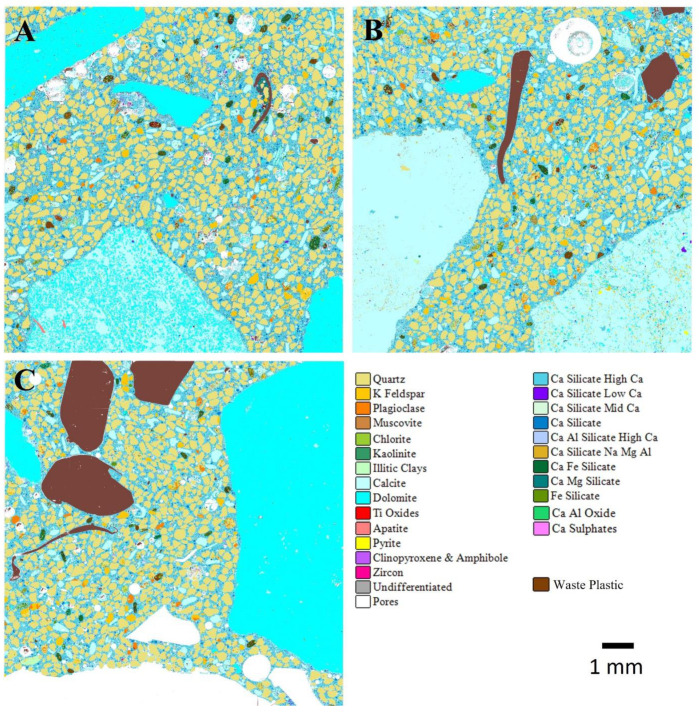
AMICS automated mineralogy false-colour images of 28-day-cured ordinary concrete mixes for (**A**) C0—control, (**B**) CWP1 with 30% WP, and (**C**) CWP2 with 70% WP.

**Figure 11:**
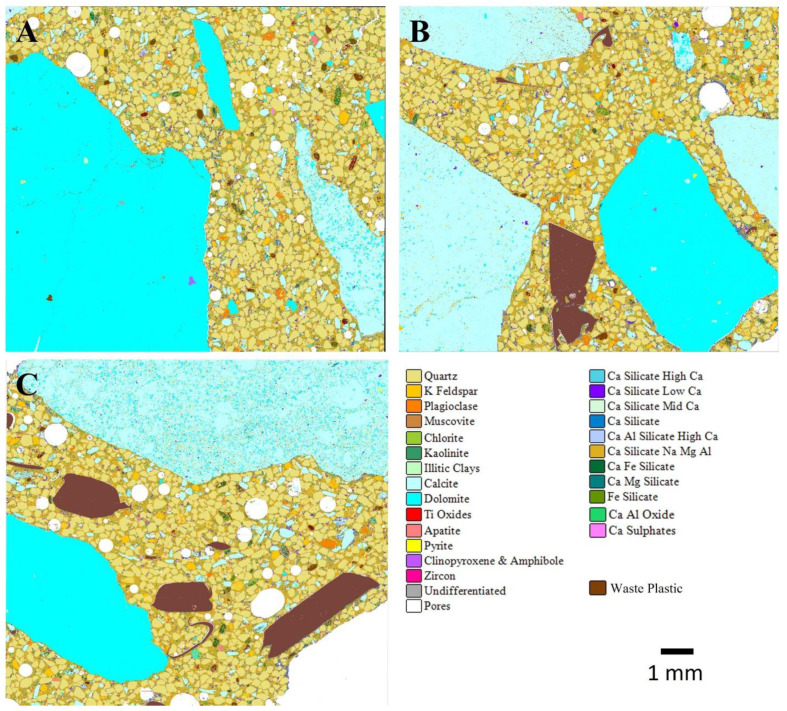
AMICS automated mineralogy false-colour images of 28-day-cured geopolymer concrete mixes for (**A**) GC0—control, (**B**) GCWP1 with 30% WP, and (**C**) GCWP2 with 70% WP.

The automated SEM-EDS (AMICS) images from slices of the dried ordinary and geopolymer concrete cube specimens with 0%, 30%, and 70% WP at 28 days of curing are shown in Figure 10 and Figure 11. Generally, observations showed a reduction in the pores for both ordinary and geopolymer concrete after 28 days of curing age. The reduction in pore sizes could be attributed to the production of the relevant cementitious hydrate (CSH and geopolymer gel) that fills up the pores to form a denser structure. The importance of the microscopic inspection of pores has been investigated by some researchers who concluded that the porosity and density of the structure are directly proportionate to the composite’s durability and mechanical performance [2,48].

Although some pores were still evident at 28 days of curing, geopolymer mixes GC0, GCWP2, and GCWP2 depict a denser and more uniform microstructure at 0%, 30%, and 70% WP and experienced higher unconfined compressive strength compared with ordinary concrete mixes.

## 5. Conclusions

The results from this study indicate the practicality and efficacy of incorporating waste plastic (polylactic acid-type) in geopolymer concrete production by replacing 10 mm of natural coarse aggregates (NA) with up to 70 wt%. The following conclusions can be drawn:

1. The consistency results indicated a gradual decline in slump values for every increase in the quantity of WP for both geopolymer and ordinary concrete mixes. This behaviour is more pronounced in geopolymer formulations due to the high viscosity of the developed geopolymer paste and the non-uniformity in the shape of WP. Therefore, any WP replacement of more than 70 wt% could impact the overall workability of the concrete produced. However, appropriate admixtures (superplasticiser) could be applied to improve the workability further.
2. A UCS comparison between geopolymer and ordinary concrete with and without the inclusion of WP, indicated superior strength development for the geopolymer concrete within the range of 46.7–58.3% at each curing age duration, indicating the robustness of the geopolymerisation reaction in concrete. Mix C0 and GC0 without WP achieved the highest UCS of 25.5 MPa and 55.4 MPa, respectively, while mix CWP2 and GWP2 with 70% WP replacement levels, performed the lowest with a UCS of 21.5 and 40.4 MPa at 28 days. In addition, the compressive strength of the ordinary and geopolymer concrete formulations declined with increasing dosages of WP (30% and 70%) due to the weak interfacial bonds between WP and the binder matrix. Moreover, this was more pronounced in ordinary concrete mixes, while the binder matrix in geopolymer concrete showed more interaction with WP.
3. An SEM analysis indicates that adding WP in both ordinary and geopolymer concrete reduced the presence of pores within the microstructure. However, this was detrimental to the ordinary concrete due to the low ITZ between the CSH gel and WP, resulting in the formation of cracks. The observation justifies the significant reduction in strength for ordinary concrete compared with geopolymer concrete. In contrast to the ordinary concrete mix, the presence of pores in the geopolymer mixes (GC0, GCWP2, and GCWP2), which reduced over the duration of the curing period, did not significantly impact the UCS due to the robustness of the geopolymer gel (Na-bearing aluminosilicates) and improved ITZ with both the WP and the natural aggregates.
4. Automated SEM-EDS quantifies the natural aggregates, waste plastics, and cementitious hydrates such as CSH and Na-bearing aluminosilicates (geopolymer) gel present.
5. Although a concrete mix with 70% WP showed a significant reduction in UCS in both concrete formulations, geopolymer concrete can still be applied to construction practice depending on the required design strength.
6. Further research can be carried out to improve the consistency and strength of geopolymer concrete incorporated with other types of plastic for its use in structural applications.

## Figures and Tables

**Table 1:** Oxide compositions of OPC, GGBS, and SF [5].

Oxides	Compositions (%)		
	Cement	GGBS	SF
CaO	61.49	37.99	0.2
MgO	3.54	8.78	0.1
SiO_2_	18.84	35.54	97.1
Al_2_O_3_	4.77	11.46	0.2
Na_2_O	0.02	0.37	-
P_2_O_5_	0.1	0.02	0.03
Fe_2_O_3_	2.87	0.42	0.01
Mn_2_O_3_	0.05	0.43	-
K_2_O	0.57	0.43	0.2
TiO_2_	0.26	0.7	-
V_2_O_5_	0.06	0.04	-
BaO	0.05	0.09	-
SO_3_	3.12	1.54	0.1
Loss on ignition	4.3	2	0.5

**Table 2:** Physical compositions of OPC, GGBS, and SF [5].

Other Properties	Cement	GGBS	SF
Insoluble residue	0.5	0.3	-
Bulk density (kg/m^3^)	1400	1200	300
Specific gravity	3.15	2.9	3.15
Glass content (%)	-	90	-
Blaine fineness (m^2^/kg)	365	450	-
Alkalinity value (pH)	13.41	10.4	7
Colour	Grey	Off-white	Grey
Physical form	Fine powder	Fine powder	Powder

**Table 3:** Some physical properties of the coarse and fine aggregates.

Physical Properties	Coarse Aggregates		Fine Aggregates (Sand)	Waste Plastics (WP)
	20 mm	10 mm		
Uniformity coefficient (CU)	1.3	3.3	0.11	-
Curvature coefficient (CC)	7.5	1.5	1.75	-
Flakiness index (%)	23	30–35	-	-
Elongation index (%)	12	17–22	-	-
Shape index (%)	7	12	-	-
Impact value	15	23	-	-
Fineness modulus (mm)	-	4	1.54	-
$\mathrm{Uncompacted}\;\mathrm{bulk}\;\mathrm{density}\;(kg/m^{3})$	2570	1350	1500	-
Pre-dried particle density $\left(kg/m^{3}\right)$	-	2690	2600	-
Water absorption (%)	1.1	2	21	-
Heat Deflection Temperature—Hdt (°C)	-	-	-	52
Density (g/cm³)	-	-	-	1.24
Tensile strength (MPa)	-	-	-	50
Flexural strength (MPa)				80
Impact strength (Unnotched) Izod (J/m)	-	-	-	96.1
Shrink rate %(in/in)	-	-	-	0.37–0.41 (0.0037–0.0041)

**Table 4:** Mix composition of ordinary concrete (C0) and geopolymer concretes (GC).

Mix Code	Elaborated Abbreviation	Concrete Binder						W (L)	Aggregates (kg)			
		OPC (kg)	Geopolymer Binder						NFA	WP	Natural Coarse Aggregate	
			GGBS (kg)	A/P	SS:SH	Activator (mL)						
				Ratio		SS	SH				10 mm	20 mm
C0	C0-OPC-0%WP-100%NA (Control 1)	3.9	-	-	-	-	-	2.2	7.9	-	3.9	7.9
CWP1	CWP1-OPC-30%WP-70%NA	3.9	-	-	-	-	-	2.2	7.9	1.2	2.7	7.9
CWP2	CWP2-OPC-70%WP-30%NA	3.9	-	-	-	-	-	2.2	7.9	2.7	1.2	7.9
GC0	GC0–0%WP-100%NA (Control 2)	-	2.6	0.5	1.2:0.8	540	360	1.1	7.9	-	3.9	7.9
GCWP1	GCWP1–30%WP-70%NA	-	2.6	0.5	1.2:0.8	540	360	1.1	7.9	1.2	2.7	7.9
GCWP2	GCWP2–70%WP-30%NA	-	2.6	0.5	1.2:0.8	540	360	1.1	7.9	2.7	1.2	7.9

OPC—ordinary Portland cement; GGBS—ground granulated blast-furnace slag; A/P—activator/precursor ratio; SS:SH—sodium silicate to sodium hydroxide ratio; natural fine aggregate—NFA; water—W; waste plastic—WP.

**Table 5:** Modal (area %) for ordinary and geopolymer concretes at 7 days of curing based on SEM-EDS automated mineral analysis.

Minerals	Area (%)					
	OPC-0%WP-100%NA	OPC-30%WP-70%NA	OPC-70%WP-30%NA	GC-0%WP100%NA	GC-30%WP70%NA	GC-70%WP30%NA
Plastic	1.58	3.52	16.28	0.23	0.19	1.91
Quartz	23.28	29.92	29.11	28.64	30.37	12.67
K Feldspar	1.04	1.30	1.05	0.95	1.54	0.53
Plagioclase	0.57	0.60	0.64	0.68	0.64	0.14
Muscovite	0.13	0.20	0.29	0.12	0.13	0.25
Biotite	0.19	0.29	0.28	0.28	0.34	0.05
Chlorite	0.26	0.28	0.29	0.27	0.34	0.06
Kaolinite	0.09	0.09	0.07	0.04	0.06	0.03
Illitic Clays	0.42	0.49	0.55	0.33	0.39	0.31
Calcite	12.50	13.76	28.42	6.22	19.59	66.97
Dolomite	34.97	17.50	4.03	33.88	16.57	8.18
Fe Oxides and Siderite	0.21	0.25	0.30	0.37	0.30	0.08
Ti Oxides	0.14	0.04	0.03	0.02	0.04	0.03
Apatite	0.09	0.01	0.00	0.00	0.08	0.01
Pyrite	0.09	0.00	0.02	0.00	0.01	0.01
Clinopyroxene and Amphibole	0.17	0.03	0.05	0.03	0.05	0.03
Zircon	0.01	0.00	0.00	0.00	0.00	0.00
Ca Silicate High Ca	2.23	2.21	1.76	0.02	0.01	0.00
Ca Silicate Mid Ca	16.87	24.16	23.47	0.52	0.75	1.03
Ca Silicate Low Ca	1.37	1.74	2.30	0.25	0.20	0.14
Ca Silicate	1.00	1.28	2.50	2.08	2.12	0.93
Ca Al Silicate High Ca	0.63	0.87	0.88	0.04	0.10	0.32
Ca Silicate Na Mg Al	0.01	0.01	0.01	21.43	23.05	6.64
Ca Fe Silicate	0.49	0.53	0.58	0.29	0.31	0.06
Ca Mg Silicate	2.12	3.04	2.26	3.26	2.70	1.42
Fe Silicate	0.14	0.14	0.13	0.19	0.20	0.08
Ca Al Oxide	0.38	0.46	0.55	0.00	0.01	0.00
Ca Sulphates	0.49	0.71	0.32	0.02	0.01	0.00
Undifferentiated	0.10	0.07	0.09	0.06	0.10	0.04

**Table 6:** Modal (area %) for ordinary and geopolymer concretes at 28 days of curing based on SEM-EDS automated mineral analysis.

Minerals	Area (%)					
	OPC-0%WP-100%NA	OPC-30%WP-70%NA	OPC-70%WP-30%NA	GC-0%WP100%NA	GC-30%WP70%NA	GC-70%WP30%NA
Plastic	2.00	2.80	10.31	0.06	3.66	6.82
Quartz	28.86	25.64	26.56	25.21	21.88	26.11
K Feldspar	1.12	1.06	1.19	0.55	0.82	1.16
Plagioclase	0.46	0.51	0.71	0.57	0.57	0.51
Muscovite	0.09	0.21	0.09	0.12	0.09	0.14
Biotite	0.25	0.26	0.14	0.29	0.18	0.30
Chlorite	0.19	0.25	0.23	0.29	0.23	0.29
Kaolinite	0.03	0.05	0.04	0.05	0.03	0.03
Illitic Clays	0.32	0.42	0.36	0.38	0.24	0.38
Calcite	17.92	38.85	7.99	8.01	32.24	28.46
Dolomite	16.32	2.64	36.35	42.66	20.08	18.72
Fe Oxides and Siderite	0.32	0.27	0.24	0.35	0.20	0.18
Ti Oxides	0.02	0.02	0.01	0.02	0.01	0.02
Apatite	0.06	0.03	0.00	0.05	0.04	0.04
Pyrite	0.00	0.02	0.00	0.00	0.02	0.01
Clinopyroxene and Amphibole	0.02	0.02	0.01	0.05	0.03	0.04
Zircon	0.00	0.00	0.00	0.00	0.00	0.00
Ca Silicate High Ca	1.94	1.78	1.21	0.01	0.01	0.01
Ca Silicate Mid Ca	24.05	22.60	19.22	0.41	0.54	0.83
Ca Silicate Low Ca	1.88	0.59	0.92	0.16	0.18	0.20
Ca Silicate	1.16	0.86	0.72	1.49	1.98	1.85
Ca Al Silicate High Ca	0.56	0.62	0.62	0.05	0.08	0.16
Ca Silicate Na Mg Al	0.02	0.01	0.01	16.50	18.20	18.11
Ca Fe Silicate	0.50	0.43	0.43	0.28	0.18	0.30
Ca Mg Silicate	2.50	1.55	1.80	2.24	2.01	1.93
Fe Silicate	0.16	0.18	0.17	0.16	0.09	0.15
Ca Al Oxide	0.45	0.40	0.49	0.00	0.00	0.00
Ca Sulphates	0.27	0.31	0.40	0.02	0.01	0.01
Undifferentiated	0.52	0.41	0.05	0.07	0.05	0.06

**Table 7:** Modal (area %) for ordinary and geopolymer concretes at 56 days of curing based on SEM-EDS automated mineral analysis.

Minerals	Area (%)					
	OPC-0%WP-100%NA	OPC-30%WP-70%NA	OPC-70%WP-30%NA	GC-0%WP100%NA	GC-30%WP70%NA	GC-70%WP30%NA
Plastic	0.10	9.89	10.10	0.19	2.47	11.30
Quartz	14.76	20.37	27.43	13.07	38.80	19.24
K Feldspar	0.57	0.76	1.07	0.53	1.54	0.76
Plagioclase	0.27	0.32	0.63	0.40	0.86	0.50
Muscovite	0.07	0.15	0.14	0.09	0.20	0.15
Biotite	0.16	0.12	0.23	0.06	0.36	0.20
Chlorite	0.15	0.11	0.21	0.11	0.32	0.24
Kaolinite	0.05	0.04	0.04	0.03	0.05	0.03
Illitic Clays	0.19	0.32	0.32	0.19	0.47	0.36
Calcite	17.06	54.96	39.58	69.77	15.90	53.76
Dolomite	50.33	0.54	0.79	2.72	2.62	1.42
Fe Oxides and Siderite	0.22	0.22	0.30	0.08	0.36	0.19
Ti Oxides	0.02	0.03	0.02	0.02	0.03	0.02
Apatite	0.01	0.00	0.01	0.01	0.02	0.01
Pyrite	0.02	0.12	0.00	0.10	0.01	0.01
Clinopyroxene and Amphibole	0.01	0.01	0.01	0.02	0.05	0.03
Zircon	0.00	0.00	0.00	0.00	0.00	0.00
Ca Silicate High Ca	0.59	1.20	1.66	0.01	0.03	0.01
Ca Silicate Mid Ca	12.03	15.72	21.44	0.86	0.82	0.97
Ca Silicate Low Ca	0.37	0.49	0.92	0.27	0.42	0.35
Ca Silicate	0.66	1.18	1.11	1.31	2.95	1.95
Ca Al Silicate High Ca	0.49	0.81	0.80	0.13	0.09	0.22
Ca Silicate Na Mg Al	0.01	0.00	0.01	8.67	29.82	17.06
Ca Fe Silicate	0.30	0.28	0.32	0.07	0.29	0.19
Ca Mg Silicate	1.04	1.31	2.00	1.34	3.61	2.09
Fe Silicate	0.07	0.09	0.10	0.06	0.27	0.12
Ca Al Oxide	0.34	0.33	0.40	0.00	0.00	0.00
Ca Sulphates	0.12	0.20	0.35	0.01	0.02	0.00
Undifferentiated	0.08	0.32	0.13	0.07	0.10	0.13

## Data Availability

Data are contained within the article.
